# Pharmacokinetic effect of disease severity and use of extracorporeal membrane oxygenation in critically ill Asian patients receiving vancomycin

**DOI:** 10.3389/fphar.2025.1506793

**Published:** 2025-02-26

**Authors:** Charul Avachat, Pi-lien Hung, Angela K. Birnbaum, Daniel P. Healy, Catherine M. Sherwin, Alex C. Lin

**Affiliations:** ^1^ Department of Experimental and Clinical Pharmacology, College of Pharmacy, University of Minnesota, Minneapolis, MN, United States; ^2^ Division of Clinical Pharmacy, Department of Pharmacy, Kaohsiung Veterans General Hospital, Kaohsiung, Taiwan; ^3^ Department of Pharmacy, School of Pharmacy, Kaohsiung Medical University, Kaohsiung, Taiwan; ^4^ James L. Winkle College of Pharmacy, University of Cincinnati, Cincinnati, OH, United States; ^5^ Internal Medicine, UWA Medical School, The University of Western Australia, Perth, WA, Australia; ^6^ Differentia Biotech Ltd., San Francisco, CA, United States; ^7^ Department of Pharmacology and Toxicology, Wright State University Boonshoft School of Medicine, Dayton, OH, United States

**Keywords:** vancomycin, pharmacokinetics, extracorporeal membrane oxygenation (ECMO), critically ill patients, methicillin-resistant *Staphylococcus aureus* (MRSA)

## Abstract

**Purpose:**

Vancomycin is an essential antibiotic for the treatment of severe gram-positive bacterial infections, including methicillin-resistant *Staphylococcus aureus* (MRSA). In critically ill patients, particularly children, attaining the appropriate dosage is crucial to avert drug resistance and ensure therapeutic efficacy. This study sought to investigate the pharmacokinetics of vancomycin in critically ill Asian pediatric patients and evaluate the influence of extracorporeal membrane oxygenation (ECMO) and disease severity on vancomycin clearance.

**Methods:**

This retrospective analysis examined data from 90 critically ill Asian patients residing in Kaohsiung, Taiwan, encompassing 263 data points gathered over 2 years. A one-compartment pharmacokinetic model with first-order elimination was constructed using nonlinear mixed-effects modeling to assess the impact of ECMO and infection severity on vancomycin clearance.

**Results:**

The pharmacokinetics of vancomycin were markedly affected by ECMO and the severity of the illness. Patients using ECMO demonstrated a 56% decrease in vancomycin clearance relative to non-ECMO patients. Furthermore, patients with milder infections (e.g., cellulitis, surgical prophylaxis, neutropenic fever) had a 39% decrease in vancomycin clearance relative to those with more severe infections (e.g., pneumonia, bacteremia, osteomyelitis, meningitis, deep tissue infection).

**Conclusion:**

The study demonstrates that ECMO and infection severity are major factors influencing vancomycin clearance in critically unwell pediatric patients. The significant decrease in clearance linked to ECMO and reduced infection severity underscores the necessity for meticulous therapeutic drug monitoring and tailored dosing strategies to enhance vancomycin treatment in this at-risk population. The findings highlight the significant interindividual diversity in vancomycin pharmacokinetics in critically unwell pediatric patients.

## 1 Introduction

Vancomycin is a frequently administered glycopeptide antibiotic that is particularly efficacious against severe Gram-positive bacterial infections, including methicillin-resistant *Staphylococcus aureus* (MRSA). Its therapeutic application is essential in pediatric populations, where accurate dosing is critical to prevent resistance and ensure efficacy. Determining appropriate vancomycin dosing regimens for critically ill pediatric patients is challenging due to significant pharmacokinetic variability, which is influenced by age, body weight, renal function, and disease severity (Stockmann et al., 2013; Keyserling et al., 2003; Xu et al., 2022). The pharmacokinetics of vancomycin exhibit considerable variability based on renal function, as demonstrated by Zaric et al. (2018), who highlighted the necessity for personalized dosing in patients with differing renal capabilities to guarantee therapeutic effectiveness and prevent toxicity.

Pediatric patients in critical condition, frequently afflicted by life-threatening ailments, face a heightened susceptibility to various infections and may necessitate pharmacological or mechanical interventions, including extracorporeal membrane oxygenation (ECMO). The utilization of vancomycin in pediatric populations has markedly increased due to the prevalence of MRSA; nonetheless, dosing continues to be intricate (Akunne et al., 2022). Therapeutic target trough concentrations for vancomycin have advanced significantly over the past decade, as subtherapeutic trough levels (5 mcg/mL) have been associated with a heightened likelihood of treatment failure (Ye et al., 2014). Vancomycin is predominantly eliminated via glomerular filtration, with approximately 90% of the administered dose excreted unchanged within 24 h, making it imperative to establish dosing regimens that take into account age, serum creatinine levels, and the susceptibility of the target organism, particularly in pediatric patients under 12 years and critically ill individuals (Shimamoto et al., 2021; Le et al., 2013). Current therapeutic medication monitoring in pediatric patients often aims for trough concentrations of 15–20 mcg/mL when managing invasive MRSA infections. Nonetheless, these targets are predominantly derived from adult studies and may have a weak correlation with pediatric outcomes (Hirai et al., 2016a). Despite these guidelines, the distinct physiological traits of juvenile patients, especially those in severe conditions, frequently require careful consideration in conventional dose recommendations. The use of ECMO, a critical intervention necessary in severe instances of infection or cardiac/respiratory failure exacerbates this. ECMO can markedly influence drug pharmacokinetics, potentially leading to suboptimal dosing if not carefully managed.

While several studies have explored vancomycin pharmacokinetics in pediatric populations, a notable gap exists in understanding the combined impact of ECMO and infection severity on vancomycin pharmacokinetics, particularly in Asian pediatric populations. For example, Stockmann et al. (2013) developed a population pharmacokinetic model for vancomycin in children with cystic fibrosis, identifying weight and renal function as significant predictors of clearance. Similarly, Akunne et al. (2022) conducted a systematic review highlighting the variability in vancomycin dosing requirements and the need for individualized therapy in critically ill children. However, these studies should have specifically addressed the influence of ECMO and infection severity on vancomycin pharmacokinetics, leaving a critical gap in the literature. This study sought to address this deficiency by delineating the pharmacokinetics of vancomycin in a critically unwell Asian pediatric cohort. It focuses on how ECMO and varying degrees of infection severity influence vancomycin clearance. By leveraging a comprehensive dataset and advanced pharmacokinetic modeling techniques, this research seeks to provide insights to inform more accurate and effective dosing strategies in this vulnerable patient group.

## 2 Materials and methods

### 2.1 Data and blood sample collection

This retrospective study used medical records of critically ill patients hospitalized in Kaohsiung Veterans General Hospital, Taiwan, from 1 January 2014, to 31 December 2016. The study was approved by the Kaohsiung Veteran General Hospital Institutional Review Board (certificate number VGHKS18-CT9-12). A waiver of informed consent was granted as the data were collected retrospectively from medical records. The identity of the study subjects was masked through the use of a study identifier. Data were de-identified. Inclusion criteria were patients receiving vancomycin for the treatment or prevention of MRSA or other bacterial infections during their hospitalization and for whom therapeutic drug monitoring had been performed. Patients were excluded if vancomycin concentrations in blood could not be detected or if there were incomplete data on the vancomycin regimen. As per the study protocol the blood samples were collected >1 h after the end of dose administration (peak concentration) and within 30 min before the next dose (trough concentration). Trough and peak concentrations obtained after administering at least four equal repeated doses were referred to as the steady-state concentrations.

Data abstracted included height, weight, age, principal diagnosis at admission, whether an open-heart surgery had been performed, serum creatinine concentrations, whether extracorporeal membrane oxygenation (ECMO), and vancomycin dosage, frequency, route, speed, and administration time indications for the use of vancomycin. The estimated glomerular filtration rate (eGFR) was calculated using the bedside Schwartz equation from serum creatinine values (Schwartz et al., 2009). There were nine distinct diagnostic categories at admission. To improve simplicity and interpretability, diagnoses were grouped into two major categories based on clinical differences, depending on the severity of the bacterial infection for which vancomycin was used. The first category (severe infections) corresponded to people diagnosed with pneumonia/bacteremia/osteomyelitis/meningitis/deep tissue infection, and the second category (mild infections) included people diagnosed with cellulitis/surgical prevention/neutropenic fever/others.

### 2.2 Pharmacokinetic analysis

Vancomycin plasma concentrations were transformed into their natural logarithms. The corresponding concentration versus time plot was placed on a logarithmic scale for all the subjects to address normality. The concentration-time profiles were characterized using nonlinear mixed-effects modeling using Phoenix NLME (Certara Inc. version 8.2). Concentrations below the lower limit of detection (LLOD) were excluded from the analysis. Concentrations below the lower limit of quantitation (LLOQ) were divided by two to obtain an unbiased conservative estimate of the true concentration. One- and two-compartment models with first-order elimination with additive, proportional, and combined error models were considered for the base model. The between-subject variability was normally distributed with a mean equal to 0 and variance equal to ω^2^. The allometric scaling exponent for clearance and volume of distribution were fixed at 0.75 and 1, respectively (Holford, 1996; Chung et al., 2021). The models were run with the FOCE-ELS algorithm and simple run mode. Model comparison was made using the Akaike information criterion (AIC) with the model producing the lowest value deemed superior. Weight was applied *a priori* to the base model. A sensitivity analysis was performed after the final structural model selection, and the initial estimates were changed to evaluate the robustness of the model.

### 2.3 Covariate investigation

The clinically relevant categorical covariates that were investigated included the major diagnosis category/infection severity, extracorporeal membrane oxygenation (ECMO) status, and surgery status. Covariate investigation was conducted in stepwise covariate search run mode in Phoenix (Phoenix^®^, version 8.2, Certara, Princeton, NJ) using stepwise forward inclusion followed by stepwise backward elimination. The models were assessed by a reduction or increase in the objective function value (OFV), equal to −2*log likelihood. During forward selection, any covariate that reduced the OFV by > 3.84 [p < 0.05, χ2 distribution with 1 degree of freedom (df)] was significant. Each covariate was then independently removed from the model, one at a time, and an increase in the OFV of >6.635 (p < 0.01, χ2 distribution with one df) deemed the covariate significant for inclusion in the final model. The full model was then constructed with significant covariates added.

### 2.4 Model evaluation

The following diagnostic plots were generated: (a) observed versus population predicted concentrations (DV vs. PRED), (b) observed versus individual predicted concentrations (DV vs. IPRED), (c) conditional weighted residuals versus time (CWRES vs. IVAR) (d) conditional weighted residuals versus population predicted concentrations (CWRES vs. PRED). The shrinkage values for the individual parameters were directly calculated from model runs. The predictive performance of the final model was evaluated using a visual predictive check (VPC; Predictive check option in Phoenix^®^ NLME). VPC employed Monte Carlo simulations to generate concentration-time profiles of 2,000 patients. The observed data’s 5th, 50th, and 95th percentiles were superimposed with the median values and the 5th,50th, and 95th percentiles of the simulated concentration-time profiles. The model was deemed precise if the concentration-time data were approximately distributed within the simulated data’s 5th to 95th prediction interval. The stability of the final model was evaluated by non-parametric bootstrap analysis on 1,000 datasets sampled from the original dataset at random with replacement. The bootstrap parameters were compared with the final model parameter estimates. The standard error, % coefficient of variation (CV), and 95% confidence interval (CI) of the bootstrap parameters were also reported.

## 3 Results

### 3.1 Study participants

**FIGURE 1:**
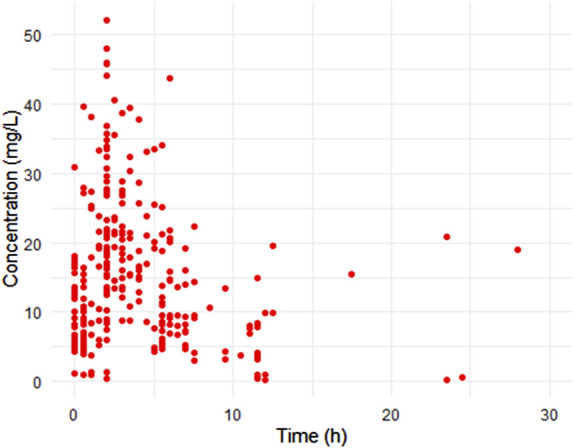
Scatter plots of observed concentration versus time after dose. Representing a total 263 samples from 90 patients.

The study included 90 patients with a median age of 3.7 years (range: 0.1–36.6 years) and weight of 12.6 kg (range: 0.7–93 kg). The median eGFR was 70.4 mL/min (2.4–150.6 mL/min). Among the patients, 12 (13.3%) were on extracorporeal membrane oxygenation (ECMO) and 33 (36.7%) had undergone surgery (Table 1). Ninety percent of the patient population comprised pediatric subjects. A total of 263 data points were used in the pharmacokinetic analysis. The infusion rate ranged from 8 to 1,500 mg/h, and there was a broad distribution in concentration and sampling time (time elapsed since the first dose) (Figure 1).

**TABLE 1:** Demographic characteristics.

Characteristic	
N	90
Age, Median (Range), years	3.7 (0.1–36.6)
Weight, Median (Range), kg	12.6 (0.7–93)
eGFR, Median (Range), mL/min	70.4 (2.4–150.6)
Sex, N	
Male	47
Female	43
Diagnoses, N^a^	
Severe infection diagnosis	
Pneumonia	20
Bacteremia	6
Osteomyelitis	7
Meningitis	4
Deep tissue infection	1
Mild infection diagnosis	
Cellulitis	6
Surgical prevention	35
Neutropenic fever	4
Others	7
Dialysis status, N	
Regular HD	1
Emergent HD	—
CRRT	1
Other	88
Indication, No.	
MRSA	4
Severe MRSA	8
Non-MRSA	27
Empirical	50
Other	1
ECMO status, N	
Yes	12
No	78
Surgery Status, N	
Yes	33
No	57

N: Number of study participants; eGFR: estimated Glomerular filtration rate; HD, Hemodialysis; CRRT, Continuous renal replacement therapy; MRSA, Methicillin-resistant *Staphylococcus aureus*; ECMO, Extracorporeal membrane oxygenation.

^a^ Combined into two categories as described under covariate investigation section.

### 3.2 Pharmacokinetic analysis

A one-compartment model was chosen due to its structural simplicity and lower AIC value [one-compartment (1842.94) and two-compartment (1856.55)]. The proportional error model (AIC value: 1842.94) performed better than an additive error model (AIC value: 1841.38) in explaining the residual variability and diagnostic plots and thus was chosen as the base error model. The final model was a one-compartment model with first-order elimination and proportional error fully described vancomycin’s pharmacokinetics. The drug’s elimination was assumed to occur from the central compartment. The model was parametrized using clearance (CL) and volume of distribution (Vd).

### 3.3 Covariate investigation and final model

Eta plots were utilized to investigate the association of clinical covariates with clearance and volume of distribution. During the stepwise covariate forward addition and backward elimination process, two covariates, namely, infection severity and ECMO status, were identified as statistically significant. The stepwise covariate search indicated that ECMO status, infection severity (diagnosis), and surgery status significantly influenced vancomycin clearance (CL). The final model included weight, ECMO status, and infection severity as key covariates retained after backward elimination (Table 2). As shown in Table 3, the clearance of vancomycin was significantly reduced in patients undergoing ECMO, with a mean clearance of 0.48 L/h (95% CI: 0.42-0.50), representing a 56% reduction compared to non-ECMO patients. Similarly, patients with less severe infections (diagnosis of cellulitis/surgical prevention/neutropenic fever/others) demonstrated a 39% reduction in clearance, with a mean value of 0.63 L/h (95% CI: 0.58-0.68). These findings indicate that both ECMO status and infection severity are major determinants of vancomycin pharmacokinetics in this population. Incorporating infection severity and ECMO status on CL significantly improved the model when added stepwise and could be retained in the backward elimination step.

**TABLE 2:** Stepwise covariate search using forward inclusion and backward elimination.

Model number	Model description	OFV (−2LL)	Δ OFV	P-value
Forward Inclusion				
1	Base model with weight on Vd and CL	1822.82		
2	Adding surgery on CL to model 1	1814.39	8.43	<0.05
3	Adding infection severity on CL to model 2	1809.78	4.61	<0.05
4	Adding ECMO status on CL to model 3	1804.9	4.88	<0.05
Backward elimination				
5	Removing surgery on CL from model 4	1806.39	5.6	>0.01

All the remaining covariates on removal had a p-value <0.01 and were thus retained in the final model.

CL, Clearance; Vd, volume of distribution; ECMO, extracorporeal membrane oxygenation; OFV, objective function value; ΔOFV, change in Objective Function Value.

**TABLE 3:** Covariate impact on clearance (CL).

Effect of extra corporeal membrane oxygenation on clearance		
	Patients with ECMO (ECMO = = 1)	Patients without ECMO (ECMO = = 0)
Equation	${Cl=tvCl*\left(\frac{WT}{median\;WT}\right)}^{dCldWT}*\exp\;\left(\text{dCldECMO }*1\right)$	${Cl=tvCl*\left(\frac{WT}{median\;WT}\right)}^{dCldWT}$
After substituting values from Table 3	$Cl=1.03*1^{0.75}*\exp\;\left(-0.81\right)$	$Cl=1.03*1^{0.75}$
Clearance value (L/h)	0.48	1.03
% difference in clearance	∼56%	

Effect of infection severity on Clearance		
	Patients with mild infections (DIAG = = 1	Patients with severe infections (DIAG = = 0)
Equation	${Cl=tvCl*\left(\frac{WT}{median\;WT}\right)}^{dCldWT}*\exp\;\left(\text{dCldDIAG }*1\right)$	${Cl=tvCl*\left(\frac{WT}{median\;WT}\right)}^{dCldWT}$
After substituting values of final PK parameters in Table 4	$Cl=1.03*1^{0.75}*\exp\;\left(-0.49\right)$	$Cl=1.03*1^{0.75}$
Clearance value (L/h)	0.63	1.03
% difference in clearance	∼39%	

Note: Calculations have been done assuming an individual with weight equal to the median weight of the population and ηCl=0 .

tvCL, is the typical value of clearance of vancomycin in the study population; dCldECMO, is the fixed-parameter coefficient of extracorporeal membrane oxygenation; dCldDiag is the fixed-parameter coefficient of the infection severity and; ηCL, is the random effect for clearance, which is representative of the variability in clearance.

The following equations represented the final model with the inclusion of significant covariates:

$$Cl=tvCl\;*\;{\left(\frac{WT}{median\left(WT\right)}\right)}^{dCldWT}*\exp\left(dCldECMO1*\left(ECMO==1\right)\right)*\exp\left(dCldDIAG1*\left(DIAG==1\right)\right)*\exp\left(\eta Cl\right)$$

where, tvCL is the typical value of clearance of vancomycin in the study population;dCldECMO is the fixed-parameter coefficient of extracorporeal membrane oxygenation;dCldDiag is the fixed-parameter coefficient of the infection severity andηCL is the random effect for clearance, which is representative of the variability in clearance.ECMO = = 1indicating patients on ECMO.DIAG = = 1 indicating a patient belongs to mild infection category

$$V=tvV\;*\;{\left(\frac{WT}{median\left(WT\right)}\right)}^{dVdWT}*\exp\left(\eta V\right)$$

where, tvV is the typical value of the volume of distribution of vancomycin in the study populationηV is the random effect for the volume of distribution, which is representative of the variability in the volume of distribution.

WT, ECMO, and DIAG represent weight, extracorporeal membrane oxygenation, and infection severity in the equations. As per the above equation, a typical individual is someone weighing 12.6 kg, which is the median weight of the study population, and having a severe infection (diagnosed with pneumonia/bacteremia/osteomyelitis/meningitis/deep tissue infection). The final pharmacokinetic model estimated a clearance (tvCL) of 1.03 L/h (95% CI: 0.84-1.23) and a volume of distribution (tvVd) of 11.61 L (95% CI: 9.2-14.03) for a typical individual (Table 4).

**TABLE 4:** Final parameter estimates and bootstrap results.

Parameter	Final model					Bootstrap			
	Estimate	SE	95% CI	%CV	Shrinkage	Median	SE	95% CI	%CV
tvV (L)	11.61	1.22	9.2–14.03	10.55	-	11.85	3.26	7.69–20.1	26.3
tvCL (L/h)	1.03	0.099	0.84–1.23	9.67	-	1.01	0.17	0.75–1.41	16.24
dCldDiag	−0.49	0.18	−0.84–−0.14	36.79	-	−0.48	0.18	−0.87–−0.13	36.18
dCldECMO	−0.81	0.17	−1.14–−0.47	21	-	−0.79	0.19	−1.19–−0.4	24.58
dCldWT (fixed)	0.75	-	-	-	-	0.75	-	-	-
dVdWT (fixed)	1	-	-	-	-	1	-	-	-
η Cl	0.26	0.08	0.1–0.42	30.7	0.27	0.25	0.09	0.07–0.43	36
η V	0.24	0.07	0.1–0.38	29.2	0.52	0.24	0.12	0–0.48	50
Stdev0	0.44	0.03	0.39–0.5	6.54	-	0.45	0.03	0.38–0.51	7.6

tvV, typical value of Vd; tvCL, typical value of CL; dCldDiag, fixed parameter coefficient of infection severity; dCldECMO, fixed parameter coefficient of extracorporeal membrane oxygenation; dCldWT, allometric scaling exponent for clearance; dVdWT, allometric scaling exponent for volume of distribution; stdev0, standard deviation; SE, standard error; CI, confidence interval; CV, coefficient of variation.

In the context of our analysis, the volume of distribution (Vd) represents the theoretical volume in which vancomycin is distributed in the body. Lower Vd values in the ECMO group suggest a more restricted distribution, potentially due to alterations in body fluid compartments during ECMO therapy.

### 3.4 Model evaluation

**FIGURE 2:**
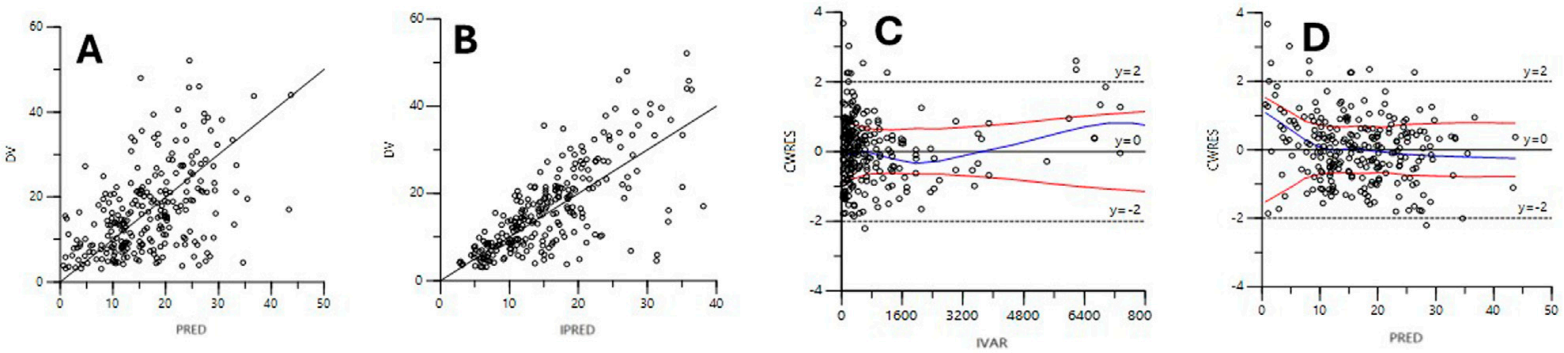
Diagnostic scatter plots of the final model. **(A)** Observed versus population-predicted concentrations (DV vs PRED); **(B)** Observed versus individual predicted concentrations (DV vs. IPRED); **(C)** Conditional weighted residuals versus time (CWRES vs IVAR); **(D)** Conditional weighted residuals versus population-predicted concentrations (CWRES vs. PRED).

**FIGURE 3:**
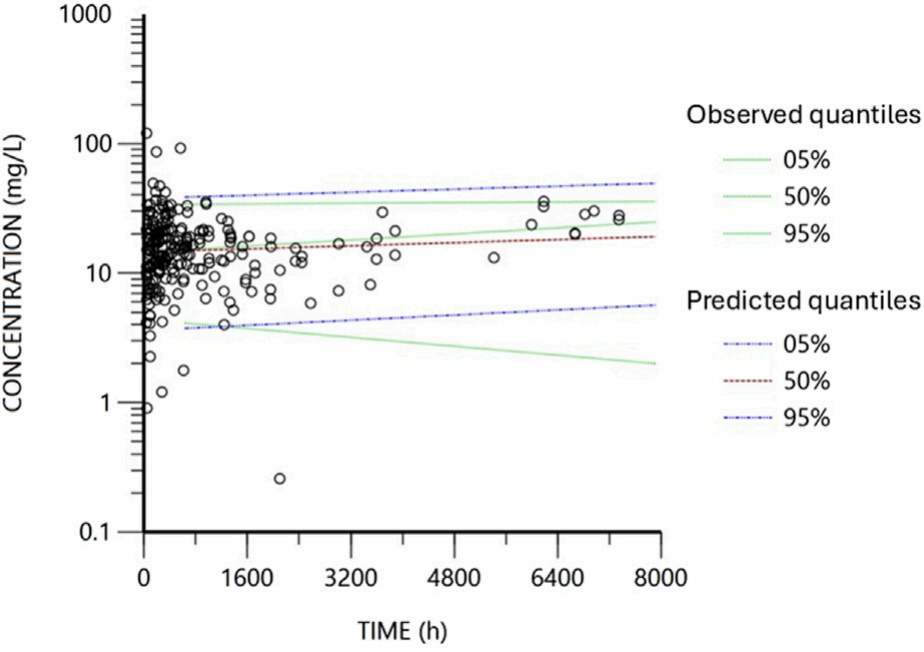
Visual predictive check obtained from 2000 simulations of the database. Black circles represent the observed concentrations. Blue lines represent the data’s predicted 5th and 95th percentiles, while red represents the predicted 50th percentile. The dotted green lines represent the observed fifth, 50th and 95th percentiles.

The diagnostic plots for the base and final models are presented in Supplementary Figure S1; Figure 2. The shrinkage factor for volume and clearance was 0.52 and 0.27, respectively, indicating individual estimates might be dominated by the population mean. The VPC in Figure 3 further confirmed the model’s predictive accuracy, with observed data closely aligning with the simulated prediction intervals. Most of the data was distributed within the 5th to 95th prediction interval, which supports the precise performance of the final model. The median bootstrap estimates were comparable to the parameter estimates from the final model. The bootstrap estimates were within the 95% confidence interval, demonstrating the stability of the model across different resampled datasets and supporting the validity of the findings.

## 4 Discussion

The findings of this study offer significant insights into the pharmacokinetics of vancomycin in critically ill pediatric patients, particularly in the context of ECMO use and varying infection severities. Our findings are consistent with previous research by Moffett et al. (2018) and An et al. (2020), which identified that ECMO therapy in neonates was associated with reduced vancomycin clearance (Chow et al., 2020).

In pediatric patients, vancomycin pharmacokinetics can be highly variable due to age-related differences in drug absorption, distribution, metabolism, and elimination. Population pharmacokinetic models provide quantitative and semi-quantitative frameworks for optimizing dosing regimens. They achieve this by identifying sources of variability, by determining factors that influence the pharmacokinetic behavior of the drug, and quantifying the magnitude of unexplained variability (Stockmann et al., 2013). Previous data show that dialysis session duration and dialysis mode (HD versus HDF) are significant predictors of pharmacokinetic parameters, and the half-life is shorter for HDF than for HD (2.1 vs 3.5 h) (Chung et al., 2021; Matusik et al., 2022). Then MRSA is suspected, and vancomycin is administered to children in intensive care units, the concentration should be monitored to prevent renal impairment (Chung et al., 2021; Matusik et al., 2022).

A population modeling method was employed in the present study enabling a thorough quantitative assessment of the impact of clinical and pathophysiological covariates on vancomycin pharmacokinetics in an Asian subpopulation. The pharmacokinetics of vancomycin were best described using a one-compartment model with first-order elimination following IV infusion. Data for this study was collected from a hospital setting utilizing therapeutic drug monitoring, which was leveraged to develop the model. Extracorporeal membrane oxygenation significantly explained a portion of the interindividual variability in clearance, which matches the findings of the literature (Moffett et al., 2018; An et al., 2020; Zylbersztajn et al., 2018). This is the first model to elicit the severity of bacterial infection as a significant covariate that aided in elucidating the differences in clearance between individuals The study revealed that conditions such as pneumonia, bacteremia, osteomyelitis, meningitis, or deep tissue infection are linked to increased vancomycin clearance, likely due to augmented renal clearance (ARC), frequently observed in critically ill patients. ARC is a complex clinical condition resulting from multiple mechanisms, where the kidneys exhibit increased clearance of drugs or substances. It is challenging to assess in pediatric critically ill patients due to the lack of reliable methods and fluctuations in GFR (Chu et al., 2016; Hirai et al., 2016b).

This study demonstrated that ECMO is associated with an 56% reduction in vancomycin clearance, while less severe infections resulted in a 39% reduction. Our findings suggest that standard dosing regimens, often extrapolated from adult studies, may not be appropriate for pediatric patients undergoing ECMO or those with less severe infections. This variability in vancomycin pharmacokinetics highlights these patients’ critical need for individualized dosing regimens. Current dosing guidelines have traditionally recommended maintaining trough concentrations of 15–20 mcg/mL to ensure therapeutic efficacy and prevent resistance, especially for invasive MRSA infections. However, our findings indicate that achieving these trough concentrations in ECMO patients, who exhibit significantly reduced clearance, may require lower doses or extended dosing intervals to avoid potential toxicity. Conversely, patients with severe infections may face the risk of subtherapeutic levels under standard dosing regimens, necessitating more aggressive dosing strategies to ensure adequate drug exposure. While vancomycin time-versus-concentration profiles have historically been described as monophasic, biphasic, or triphasic, with most literature supporting a biphasic process, there is growing recognition that trough concentrations alone may not adequately reflect therapeutic exposure. For instance, variations in the steady-state volume of distribution (ranging from 0.39 to 2.04 L/kg depending on age, gender and body weight (Hirai et al., 2016b). This complexity underscores the need for more precise dosing strategies. Le et al. (2013) demonstrated that using the area under the curve (AUC) exposure for vancomycin dosing in children significantly improved target attainment compared to traditional trough-based monitoring. Reflecting this evidence, the ASHP/IDSA guidelines from 2020 now recommend AUC-guided dosing and monitoring as a more accurate and safe approach for vancomycin administration. Such AUC-guided methods may be particularly beneficial for patients undergoing ECMO or those with varying infection severities, as they allow for more individualized and clinically relevant dosing adjustments based on specific patient characteristics.

Reports from clinicians who work in the neonatal or pediatric intensive care units (NICU or PICU) of 12 major medical centers in Hong Kong found that renal function, post-menstrual or postnatal age, body weight, and suspected or documented pathogens were cited as the most significant clinical parameters for determining the initial vancomycin dosage (Stockmann et al., 2014). Respondents cited difficulties in determining the optimal initial dose for a targeted level, inconsistencies between dosing references, and the absence of clear hospital guidelines as challenges to optimal dose adjustment (Hirai et al., 2016a; Hirai et al., 2016b).

Given the variability in vancomycin pharmacokinetics, therapeutic drug monitoring (TDM) emerges as an essential tool for managing vancomycin therapy in critically ill pediatric patients. TDM allows for real-time adjustments to dosing regimens, ensuring each patient receives a dose tailored to their specific pharmacokinetic profile (Yu et al., 2014). This personalized approach is particularly crucial in high-risk populations, such as those undergoing ECMO, where the margin for error is narrow, and the risk of toxicity is high. The study’s findings suggest that for ECMO patients, a lower initial vancomycin dose may be appropriate, with close monitoring of serum concentrations to guide subsequent dosing adjustments. Conversely, patients with severe infections may benefit from more frequent dosing or higher doses to maintain therapeutic drug concentrations. Regular TDM ensures that dosing can be adjusted dynamically, minimizing the risks of both underdosing (leading to treatment failure) and overdosing (leading to toxicity). This study’s substantial variability in vancomycin pharmacokinetics underscores the need for individualized dosing strategies in critically ill pediatric patients. This aligns with the findings of Akunne et al. (2022), who conducted a systematic review highlighting the variability in vancomycin dosing requirements and the need for individualized therapy in critically ill children.

## 5 Study limitations

This study provides valuable insights into the pharmacokinetics of vancomycin in critically ill pediatric patients, but several limitations must be acknowledged. The study’s retrospective design is limited by the availability and quality of existing medical records, which could introduce bias and limit the accuracy of the pharmacokinetic model. The classification of different diagnoses into severe and mild infections in this study was based on clinical judgment and the context of treatment, as outlined in the methods section. Neutropenic fever, categorized under the “mild infection” group, is a condition known to exhibit significant variability in vancomycin clearance, often driven by augmented renal clearance (ARC). For instance, previous studies, such as Hirai et al. (2016a), have reported higher clearance rates in these patients due to hyperfiltration. However, in the present study, only four patients were classified as having neutropenic fever, limiting the power to draw definitive conclusions about this subgroup clearance rates. The observed lower clearance in neutropenic fever patients in this study contrasts with broader literature and may reflect confounding factors. These factors include variability in the severity and progression of sepsis, the small sample size, and the potential influence of ECMO on this subgroup’s clearance rates.

One of the more unusual findings of this study was the lack of significance for GFR as a covariate influencing vancomycin clearance in the final model. While vancomycin is primarily renally eliminated, as demonstrated in the literature (e.g., Zaric et al. (2018), the inclusion of ECMO as a significant covariate may have overshadowed the renal function’s impact. ECMO is known to affect pharmacokinetics profoundly by altering fluid dynamics and renal perfusion, potentially confounding the expected relationship between GFR and clearance. Moreover, the dataset’s reliance on estimated GFR (eGFR) using the Schwartz equation may have introduced variability, especially in patients with fluctuating renal function. The median eGFR in our cohort was 70.4 mL/min/1.73 m^2^, indicating that most patients were not in renal failure. This relatively normal renal function range could have reduced the discriminatory power of GFR as a covariate. This highlights the need for close TDM to navigate these overlapping factors and ensure optimal dosing.

The study was conducted at a single medical center, which may limit the generalizability of the results due to differences in patient population, hospital practices, and regional factors at Kaohsiung Veterans General Hospital in Taiwan. Due to genetic, physiological, and environmental differences, pharmacokinetic parameters can vary significantly between ethnicities. Therefore, caution should be exercised when applying these results to non-Asian populations.

Another limitation is the high shrinkage factor observed for the volume of distribution, suggesting that the individual estimates for this parameter are heavily influenced by the population mean, likely due to sparse sampling or limited information in the data to precisely estimate individual values. This may reduce the model’s ability in predicting vancomycin pharmacokinetics in populations with different characteristics, which is particularly relevant in pediatric populations, where blood sampling is often restricted due to ethical and practical concerns. A relatively high residual error of 44% was observed, indicating that a significant portion of the variability in the observed data remains unexplained despite the inclusion of all available covariates. While all known and measurable covariates were included in the model, it is possible that additional, unmeasured factors, such as unobserved patient characteristics or complex pharmacokinetic/pharmacodynamic relationships, may contribute to the unexplained variability. Additionally, the model assumes a constant proportional error, which may not fully account for all sources of measurement or process noise in the data. However, due to data limitations and constraints in the study design with a majority of trough sample collection, it was not possible to explore alternative error models or further refine the model structure. This could affect the precision and reliability of predictions. The exploratory nature of this analysis necessitates a cautious interpretation of findings, particularly given the borderline statistical significance observed for some covariates.

Vancomycin pharmacokinetics has been described by either a one or two compartment model. The predominance of trough-level samples restricted the ability to characterize distribution phases and evaluate multi-compartmental kinetics accurately. There is thus a possibility that a two-compartment model was not able to capture the data and reach the global minimum during convergence. Additionally, the rejection of a two-compartment model in this study likely reflects boundary constraints. There are also limitations in terms of using visual predictive check for therapeutic drug monitoring data. While VPC’s offer a general idea on model performance they may not be able to adequately assess whether the drug concentrations fall within a specific therapeutic range, a critical aspect of TDM.

## 6 Future studies

Prospective studies with more controlled and standardized data collection would help mitigate certain issues highlighted in the previous section and provide more robust findings. Studies should include multiple centers with diverse patient populations to validate the findings and ensure broader applicability. Future studies should either classify neutropenic fever as a distinct group or include it under severe infections for better discrimination. Studies should also explore alternative or direct markers for ARC metrics. The absence of GFR as a significant predictor may reflect specific pharmacokinetic behaviours in critically ill pediatric patients on ECMO. This warrants further investigation to distinguish intrinsic renal effects from ECMO-related alterations. Analyses should explore the interaction between ECMO status and renal function to evaluate the possibility of multicollinearity between the two variables. Sparse sampling and limited representation of extreme renal function may have restricted our ability to identify GFR as a significant covariate. Therefore, we suggest that retrospective studies with more granular renal data and broader patient cohorts could help elucidate this relationship.

Future studies incorporating peak and mid-interval samples would enable more robust modeling of vancomycin’s pharmacokinetics and improve the evaluation of multi-compartment models. Studies should also consider evaluating the impact of concomitant medications, fluid status, or nutritional factors on vancomycin pharmacokinetics in pediatric patients.

Future research should focus on refining dosing guidelines for vancomycin in pediatric patients on ECMO, potentially through advanced pharmacokinetic models and simulations. Moreover, further investigation into the pharmacodynamics of vancomycin in pediatric patients could provide a more comprehensive understanding of the relationship between drug exposure and clinical outcomes, ultimately leading to more precise dosing recommendations.

## 7 Conclusion

In conclusion, this study emphasizes the need for personalized vancomycin dosing in critically ill pediatric patients, particularly those undergoing ECMO or with less severe infections. The significant variability in vancomycin clearance observed in this study suggests that standard dosing guidelines may need to be revised for these populations. Therapeutic drug monitoring plays a crucial role in ensuring that dosing regimens are tailored to each patient’s needs, optimizing therapeutic outcomes, and minimizing the risk of toxicity. As we move towards more personalized approaches in pediatric pharmacotherapy, these findings should inform the development of more refined dosing guidelines that better reflect the unique pharmacokinetic profiles of critically ill pediatric patients.

## Data Availability

The datasets presented in this article are not readily available due to the policies of use associated with Kaohsiung Veteran General Hospital. Data were used under agreement with Kaohsiung Veteran General Hospital for the current study and are not publicly available. Requests to access the datasets should be directed to Alex C. Lin lina@ucmail.uc.edu.

## Abbreviations

AIC, Akaike Information Criterion; ARC, Augmented Renal Clearance; ASHP, American Society of Health-System Pharmacists; BSV, Between-Subject Variability; CI, Confidence Interval; CL, Clearance; CRRT, Continuous Renal Replacement Therapy; CV, Coefficient of Variation; eGFR, Estimated Glomerular Filtration Rate; ECMO, Extracorporeal Membrane Oxygenation; HD, Hemodialysis; HDF, Hemodiafiltration; IDSA, Infectious Diseases Society of America; IV, Intravenous; MRSA, Methicillin-Resistant *Staphylococcus aureus*; OFV, Objective Function Value; PK, Pharmacokinetics; TDM, Therapeutic Drug Monitoring; Vd, Volume of Distribution; VPC, Visual Predictive Check.
