# Supplementary figures and images for: Pharmacokinetic effect of disease severity and use of extracorporeal membrane oxygenation in critically ill Asian patients receiving vancomycin
Source: Front Pharmacol. 2025 Feb 26;16:1506793. doi: 10.3389/fphar.2025.1506793 (PMC11897554; doi:10.3389/fphar.2025.1506793)

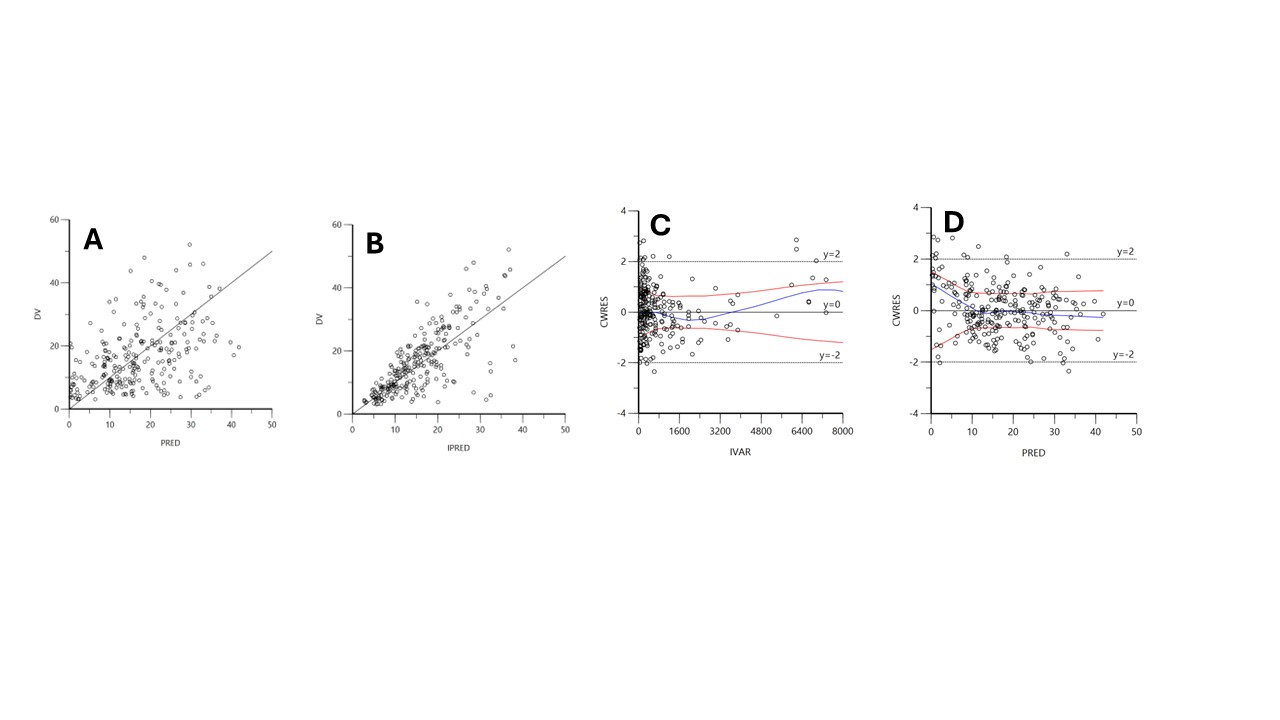

Supplement: Supplementary file 1 [file Image1.jpg]
